# Supplementary material for: The Immobilization of Hyaluronic Acid in 3D Hydrogel Scaffolds Modulates Macrophage Polarization
Source: Adv Biol (Weinh). 2025 Oct 13;9(12):e00682. doi: 10.1002/adbi.202400682 (PMC12712756; doi:10.1002/adbi.202400682)
Supplement: Supplementary file 1 — Supporting Information [file ADBI-9-e00682-s001.docx]

**Supporting Information**

**The Immobilization of Hyaluronic Acid in 3D Hydrogel Scaffolds Modulates Macrophage Polarization.**

Tiah CL Oates^1^, Jasmin Boyd^1^, Louise Dolan^1^, C de Kergariou^2^, Jingwen Zhu^3^, Ash Toye^1^, Adam W Perriman^1,3^, and Asme Boussahel^1*^

^1^School of Cellular and Molecular Medicine, Biomedical Sciences Building, University of Bristol, Bristol, BS8 1TD, United Kingdom

^2^ Bristol Composites Institute, School of Civil, Aerospace and Design Engineering (CADE), University of Bristol, University Walk, Bristol BS8 1TR, UK

^3^ Research School of Chemistry and John Curtin School of Medical Research, Australian

National University, Canberra ACT2601, Australia

***Correspondence:**

Dr. Asme Boussahel

Tel: 44 (0)117, Email: asme.boussahel@bristol.ac.uk

**Keywords: Macrophage, Hyaluronic acid, polarization, collagen, hydrogel, 3D model**

Supplemental Figures

**Supplemental Figure 1: The leeching of HA from 3D collagen hydrogel scaffolds.** a) The mean cumulative concentration of 1.5 MDa fluorescein isothiocyanate (FITC)-labelled HA in media taken from hydrogels at 24, 48, 72, and 96 hours post hydrogel synthesis. Mean and SD shown, (N=3) b) Area under the curve analysis of (a). Mean and SD shown, (N=3, ns p≥ 0.05) two-tailed t-test. c) The mean cumulative concentration of 50 kDa fluorescein isothiocyanate (FITC)-labelled HA in media taken from hydrogels at 24, 48, 72, and 96 hours post hydrogel synthesis. Mean and SD shown, (N=3) d) Area under the curve analysis of (c). Mean and SD shown, (N=3, * p<0.05) two-tailed t-test.

***
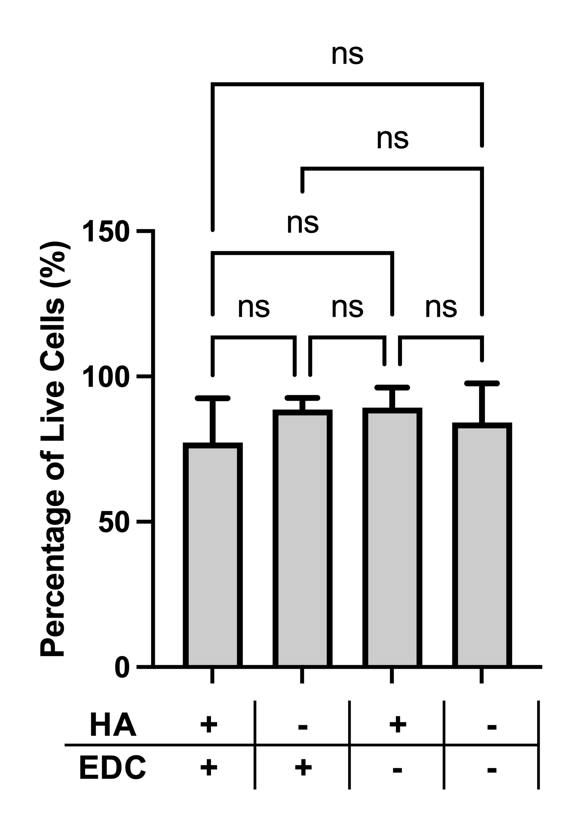
***

**Supplemental Figure 2: EDC-crosslinking of collagen hydrogels does not impact macrophage viability.** Viability of macrophages assessed by calcein AM/ ethidium homodimer immunofluorescence. Min to max boxplot displaying the mean and SD, (N=3, ns p≥ 0.05) Ordinary one-way ANOVA with Tukeys multiple comparisons test.

**Supplemental Figure 3: Macrophages express hyaluronic acid receptors CD44 and TLR4.** Percentage positivity expression of CD44 and TLR4 on macrophages as determined using flow cytometry. Min to max boxplot displaying the mean and SD, (N=3).

**Supplemental Figure 4: Rheological characterization of collagen-HA hydrogel** at 2.5 mg/ml of collagen with/without 0.2 mg/ml HA (1.5 MDa) and crosslinked or not-crosslinked with 20mM EDC. The graph **illustrates strain dependence in oscillating strain sweeps showing storage modulus G′ (full ) and loss modulus G″ (empty ),** modulus shown as log10 values**.** The addition of EDC increases both the loss and storage modulus of the collagen hydrogel for the entire oscillation strain range. It highlights an increased stiffness of the material whether the later contains HA or not. For instance the crosslinking increases 1.4*10^4% and 5.9*10^3% the storage and loss moduli of the -HA hydrogel, respectively at an oscillation strain of 50 %. It also increases 1.2*10^4% and 8.8*10^3% the storage and loss moduli of the +HA hydrogel, respectively at an oscillation strain of 50 %.

**Supplemental Figure 5 (S5):** FRAP analysis of the diffusion of FITC-dextran (3 kDa and 2000 kDa) across three hydrogel (2.5 mg/ml collagen + HA, crosslinked with EDC). (A) Time-lapse confocal images of the stages of FRAP experiment. (B) the radius of the ROI bleached. (C) Normalised fluorescence intensity recovery over time for two dextran molecular weights investigated. (D) half-life (T_1/2_) of recovery. (E) Percentage recovery. Hydrogels were prepared using 1.5 MDa HA at 200 mg/ml and crosslinked with 20 mM EDC. Mean and SD shown, (N=3, ns p≥ 0.05), Unpaired T-test. FITC-dextran at 100 mg/ml was used and incubated with the hydrogels overnight and removed before FRAP analysis.
